# Recurrent Genital Herpes Triggering Ulcerative Pyoderma Gangrenosum

**DOI:** 10.7759/cureus.40953

**Published:** 2023-06-25

**Authors:** Shreya Deoghare, Shailya Gupta, Devayani Pol, Anuja Masare, Ajay Kumar

**Affiliations:** 1 Department of Dermatology, Venereology, and Leprosy, Dr. D. Y. Patil Medical College Hospital and Research Centre, Dr. D. Y. Patil Vidyapeeth, Pune, IND

**Keywords:** herpes simplex virus type 2 (hsv-2), herpes simplex virus, unhealing ulcers, genital ulcers, genital herpes, pyoderma gangrenosum

## Abstract

Pyoderma gangrenosum (PG) is a neutrophilic dermatosis that presents as painful, rapidly growing skin ulcers with undermined edges and a violaceous, ragged border at the periphery and is non-responsive to conventional treatments. The average onset age is in the fourth decade, with a female preponderance. Genital PG is uncommon and may present singly or coexist with common sexually transmitted genital ulcerative diseases, which causes delays in the diagnosis and treatment of genital PG, thereby adding to the morbidity. Here, we highlight a case of non-healing genital ulcers that did not respond to conventional antibiotic treatment and aggravated each month with menstruation. In this case, menstruation acted as a trigger factor for the development of a herpes genital infection. The latter acts as a pathergy for the monthly aggravation of genital PG. The patient responded to treatment with anti-viral medications and immunosuppressive medications.

## Introduction

Pyoderma gangrenosum (PG) is a non-infectious neutrophilic dermatosis that presents as painful, rapidly growing skin ulcers with undermined edges and a violaceous, ragged border at the periphery [1]. The average onset age is in the second to sixth decade, with a female preponderance. It most commonly appears on the lower legs [1,2]. Due to its non-infectious pathogenesis, it is non-responsive to conventional antibiotic treatments. However, it responds rapidly to immunosuppressive medications like corticosteroids.

PG uncommonly presents in the genital areas and may present singly or in coexistence with common sexually transmitted genital ulcerative diseases. It can mimic various other causes of non-healing ulcers, like sexually transmitted diseases (syphilis, donovanosis, genital herpes, etc.), infectious diseases like tubercular ulcers, vascular causes (like vasculitis ulcers, arterial or venous ulcers), and others [2,3]. Such patients first consult a general surgeon or a gynecologist. Unawareness about this condition and the trigger factors for pathergy can cause delays in the diagnosis and treatment of genital PG, thereby adding to morbidity.

Hence, we present the case of non-healing genital ulcers, which did not respond to conventional antibiotic treatment and aggravated each month with menstruation. In this case, menstruation acted as a trigger factor for the development of a herpes genital infection. The latter acts as a pathergy for the monthly aggravation of genital PG. The patient responded to treatment with anti-viral medications and immunosuppressive medications.

## Case presentation

A 33-year-old married woman, with no history of high-risk sexual behavior and one sexually active partner (her husband), presented with complaints of multiple painful raw areas over her genitals for six weeks. She gave a history of multiple yellowish-raised lesions on the groin that formed raw areas within three to four days and were associated with severe pain.

**Figure 1:**
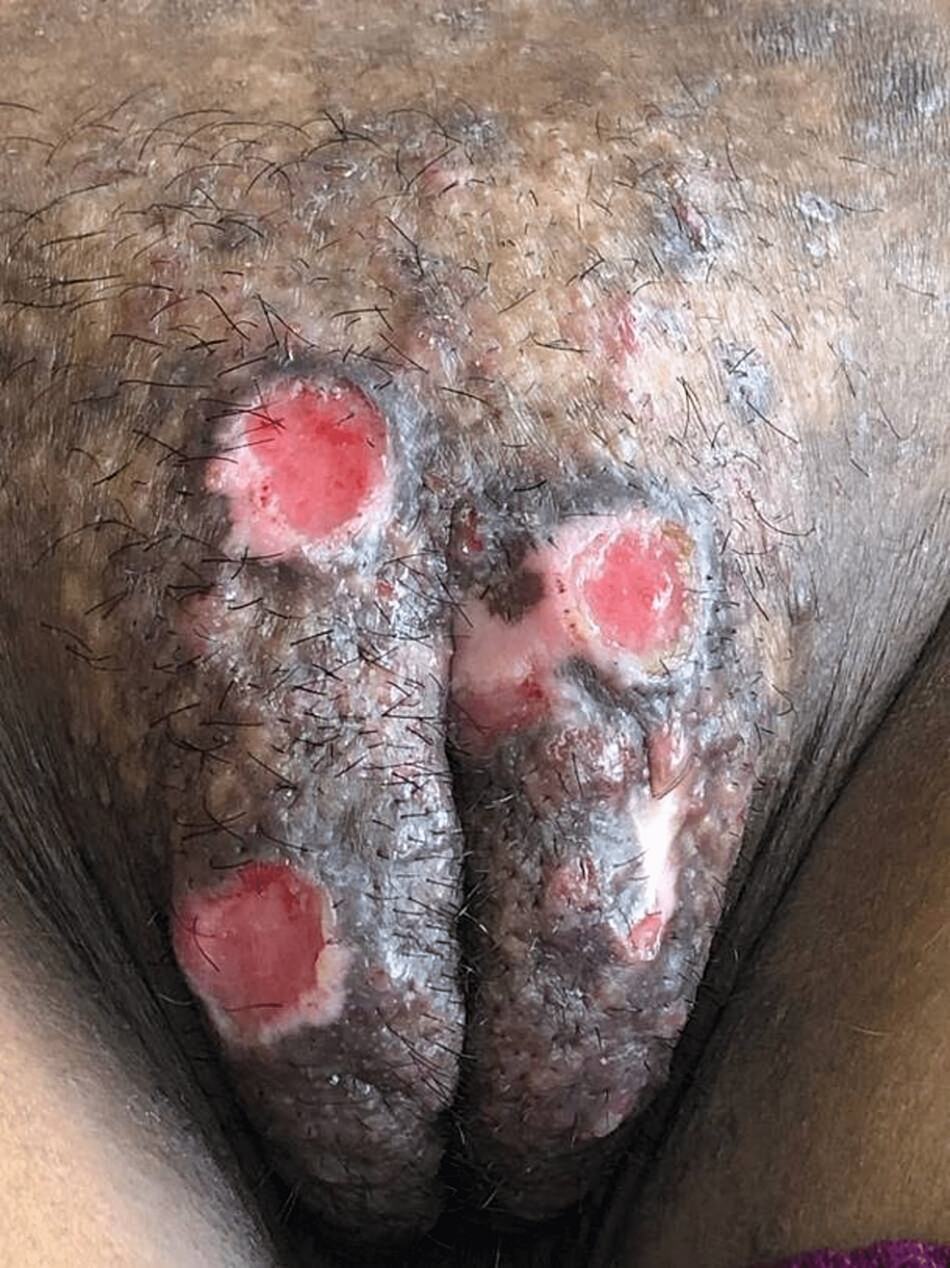
Three well-defined shallow ulcers with violaceous margins and granulation tissue at the base, one on the mons pubis and two on the vulva.

On examination, three well-defined shallow ulcers with violaceous margins and granulation tissue at the base were present, one on the mons pubis and two on the vulva (Figure 1).

**Figure 2:**
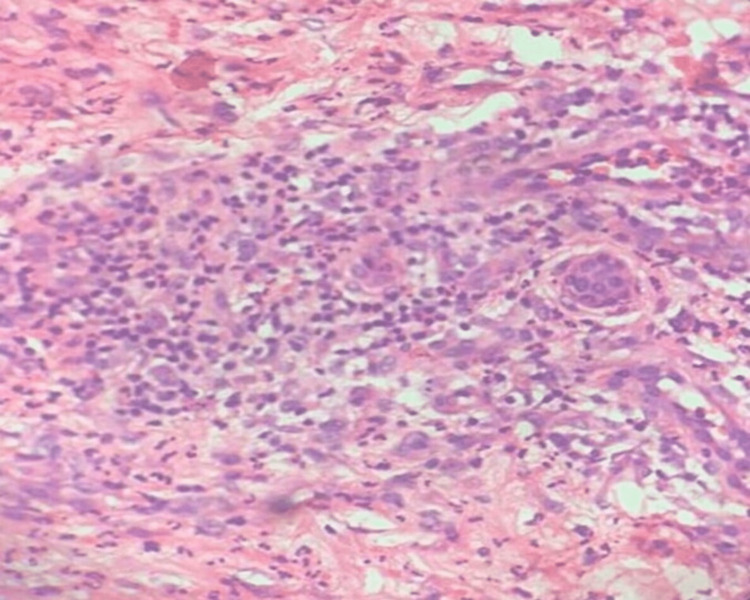
Histopathology shows a dense perivascular neutrophilic infiltrate (hematoxylin and eosin, 40x).

Histopathological examination showed a dense perivascular neutrophilic infiltrate (Figure 2).

Hematological and biochemical investigations were within normal limits, and pus cultures showed no growth. Viral markers were non-reactive. On the basis of Delphi criteria, a diagnosis of genital ulcerative PG was made [4].

The patient's initial treatment was intra-lesional triamcinolone acetate, administered once every three weeks and three times in total. While on treatment, she complained of an increase in burning and pain that aggravated with menstruation for two months. She was then started on oral cyclosporine 100 mg twice daily and topical tacrolimus 0.1% ointment twice daily. But even after this, the monthly aggravation continued, even after the initial response to the treatment.

**Figure 3:**
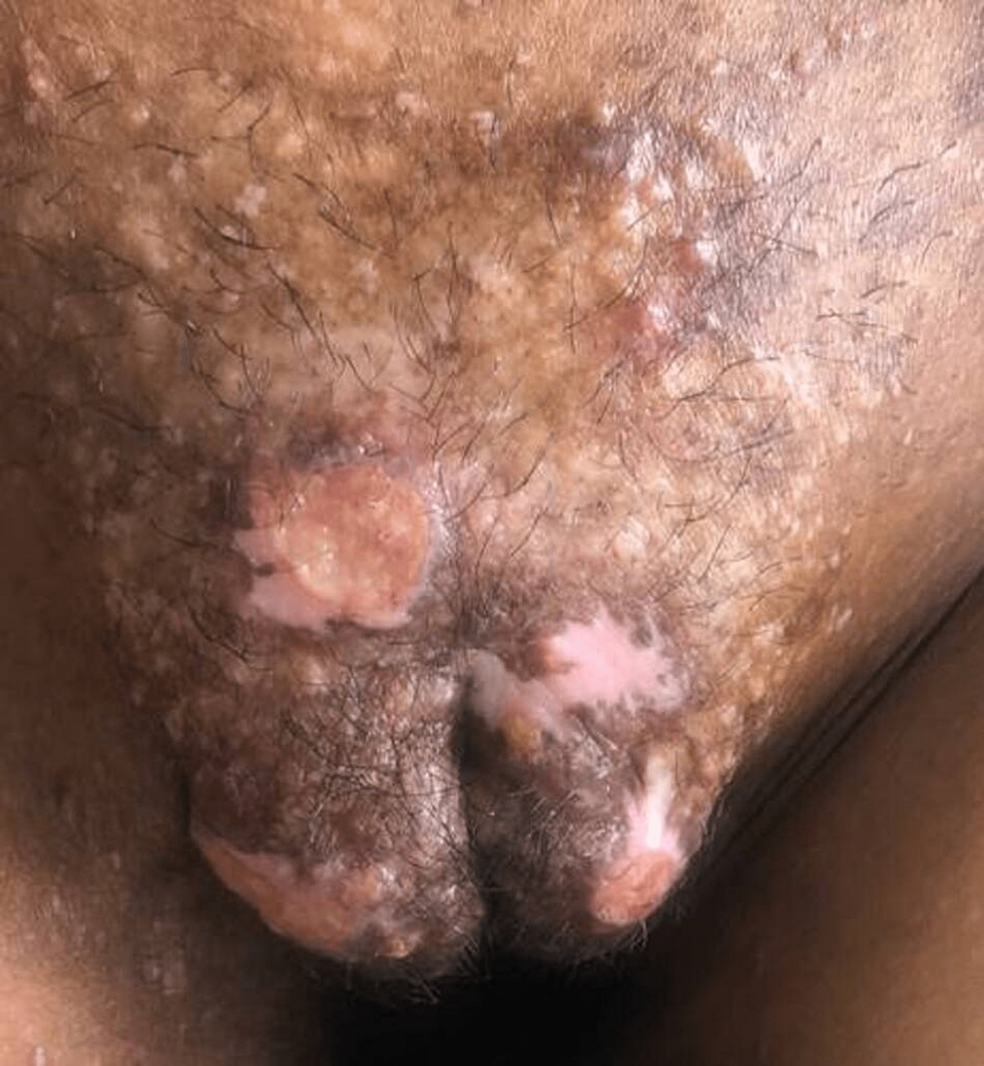
Resolving lesions after one month of treatment.

The patient was then diagnosed with genital herpes triggering before the onset of menses. This was treated initially with episodic treatment with oral acyclovir 400 mg thrice a day for five days. This was done for two months. After observing a favorable response to this treatment, the patient was shifted to a suppressive dose of oral acyclovir 400mg twice a day, along with continued oral cyclosporine and topical tacrolimus. The patient responded to this treatment (Figure 3). The herpes simplex virus 2 (HSV-2) IgG was found to be elevated.

## Discussion

Pyoderma gangrenosum is a chronic inflammatory skin condition in which a painful papule progresses to form an ulcer with undermined margins [5]. PG causes ulcerative lesions that typically affect the lower limbs but can also affect the face, penis, and vulva [6].

The diagnosis in our case was based on the Delphi criteria [4]. This criterion is exceptionally useful in its capacity to diagnose pyoderma gangrenosum using distinct major and minor criteria, as opposed to maintaining PG as a diagnosis of exclusion [4]. In our case, the biopsy from the ulcer edge demonstrated neutrophilic infiltrate, and the pus culture showed no growth. The patient gave a history of pustules that ulcerated within three to four days and healed with cribriform scarring within one month of initiating oral cyclosporine 100 mg. Therefore, our case met one major criterion and four out of eight minor criteria.

PG is commonly associated with inflammatory bowel disease, rheumatoid arthritis, and hematologic malignancies [7]. An associated underlying systemic condition is present in around 63% of patients [6]. In our case, no evidence of these associations was found when the patient presented to us.

Corticosteroids and/or cyclosporine are frequently used as the first step in the treatment of PG to reduce inflammation, followed by the addition of slower-acting immunosuppressive medications with better adverse event profiles, such as biologics [1]. In our case, the patient responded well to cyclosporine.

Genital PG is uncommon and sometimes misdiagnosed. Therefore, it should be taken into account when treating refractory genital ulcers [3]. Clinically, Behcet's disease, metastatic Crohn's disease, pemphigus vegetans, syphilis, and fungal and mycobacterial infections should all be distinguished from vulvar PG [3].

In each patient with PG with a hematological malignancy or compromised immunity, a thorough investigation for a viral agent should be conducted, particularly if the lesions are on the face or genital area [8]. Our case was associated with recurrent genital herpes, which could have been a triggering factor for PG. This emphasizes the occurrence of genital PG with viral infections.

The association between PG and HSV has been infrequently reported in the literature [9]. The first instance was reported by Wahba et al. in 1979 and involved a chronic lymphocytic leukemia patient who had HSV-2 isolated from genital PG [8]. Additionally, Tay et al. described a case of resistant PG exacerbated by herpes simplex virus infection in 2005 [10].

The monthly aggravation of the genital PG ulcers with menstruation, even while on treatment, acted as a challenge. On close examination, grouped vesicles and ulcerations were noted on the labia majora, which led us to think about a genital herpes infection triggering the PG. Subsequently, IgG for HSV-2 was also reported to be elevated. We postulate that menstruation acted as a stressor for the development of recurrent genital herpes infection, which acted as a pathergy phenomenon for the monthly aggravation of the genital PG.

## Conclusions

Recurrent herpes virus infection can act as a trigger factor for the pathergy phenomenon that leads to the aggravation of pyoderma gangrenosum (PG). Timely identification of herpes simplex and an early start of treatment can prevent the development of pathergy.
